# Fast Design Optimization and Comparative Analysis for Linear Permanent Magnet Motor with Magnet Skew, Auxiliary Tooth and Overhang Structure

**DOI:** 10.3390/s22197568

**Published:** 2022-10-06

**Authors:** Ki-Hoon Kim, Dong-Kyun Woo

**Affiliations:** Department of Electrical Engineering, Yeungnam University, Gyeongsan 38541, Korea

**Keywords:** auxiliary tooth, linear permanent magnet motor, magnet skew, overhang structure effect, virtual air-gap section method

## Abstract

This paper presents a fast design optimization using an effective characteristic analysis for linear permanent magnet motors (LPMMs) with techniques for improving motor performance such as using an auxiliary tooth, permanent magnet (PM) skew, and overhang structures. These techniques have different effects on the characteristics of the LPMM depending on the combinations of each other, resulting in complexity in the design optimization process. In particular, the three-dimensional (3-D) effect of the PM skew and overhang structure takes a lot of time to be analyzed. To deal with this problem, an effective magnetic field analysis method and a novel optimization algorithm are proposed. Preferentially, the field reconstruction method is used for a fast and accurate evaluation of the magnetic field of the LPMM. In the proposed magnetic field analysis method, the change of magnetic field distribution due to the addition of an auxiliary tooth is predicted, and the 3-D magnetic field effect of PM skew and overhang structure is considered. By reducing the computational burden in the magnetic field analysis, the electromagnetic characteristics of LPMMs can be calculated quickly, such as detent force, end force, thrust force, and back-EMF. The effect of the auxiliary tooth and overhang structure on the optimal PM skew length is investigated with comparative study results. Subsequently, the proposed optimization algorithm has the advantage of reducing time cost by providing multimodal optimization and robustness evaluation of local peaks at the same time. The proposed method is verified via comparison with finite element analysis and experimental results.

## 1. Introduction

Linear permanent magnet motors (LPMMs) have been widely used in a variety of applications where linear motion is required. The LPMMs have the advantage of preventing the mechanical loss caused by using a rotating machine as it is not necessary to convert rotational motion into linear motion. However, since they have different lengths of stator and mover, there is an end effect that causes end force [1,2]. The end effect is the leakage of magnetic flux in the end region of the back-iron, and the force generated by this is the end force. Therefore, in order to analyze the detent force in the LPMM, not only the slot effect force, which is the interaction between the slot and the permanent magnet (PM), but also the end force should be considered. The detent force that contributes to the ripple factor of thrust still remains an essential consideration for maximizing thrust in LPMMs as well as rotating machines [3].

The previous research related to the detent force reduction in the LPMM has focused on the addition of an auxiliary tooth and PM skew [4,5,6]. Seo et al. [7] and Kim et al. [8] proposed adding an auxiliary tooth to reduce the detent of the LPMM and adjusted its thickness and position. Although the detent force is reduced due to the auxiliary tooth, the air-gap magnetic flux distribution is distorted [9]. Shin et al. [10] introduced the insertion of aluminum between the back-iron and the auxiliary tooth to prevent the characteristic distortion caused by the addition of the auxiliary tooth. Kim et al. [11] compared the characteristics of the model with and without PM skew applied. However, it is difficult to find studies analyzing the characteristics of the combination between an auxiliary tooth and PM skew. Furthermore, because of the end force, the optimal PM skew in the LPMM cannot be calculated as the least common multiple of the number of slots and the number of poles used in conventional rotating machines.

**Figure 1:**
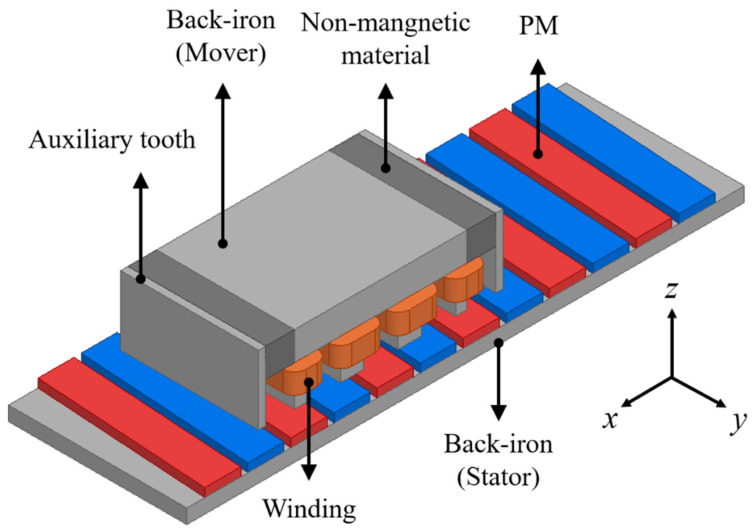
LPMM.

Figure 1 shows the LPMM with the auxiliary tooth, PM skew, and overhang structure. At the stack end of the LPMM, the magnetic flux density is relatively low due to flux leakage, resulting in a decrease in the overall air-gap magnetic flux density [12]. The overhang structure is a technique to compensate for the reduced magnetic flux density by increasing the PM length of the stator, contributing to the improvement of motor performance [13]. However, since this overhang structure is saturated with performance improvement as the PM increases, it is important to find the best compromise [14]. On top of that, this paper introduces the effect of the overhang structure on the optimal PM skew length as well as the auxiliary tooth and provides a comparative analysis.

In Figure 1, the use of three-dimensional (3-D) finite element (FE) analysis is unavoidable to analyze the characteristics of the LPMM with 3-D parts such as the PM skew and overhang structures. Although the 3-D FE analysis provides high accuracy, it is computationally expensive to handle large amounts of information. In particular, in iterative processes for design optimization, using 3-D FE analysis can be inefficient due to the significant computational burden. This paper presents an effective method for magnetic field analysis of 3-D structures. The proposed technique is based on the field reconstruction method (FRM) [15,16,17]. Using the basis function of the FRM, the entire magnetic field is reconstructed by sweeping the reference magnetic flux distribution obtained from the static FE analysis. It can predict the magnetic field at any excitation current or position condition of the mover. This results in significant time cost reduction because the conventional 3-D transient FE analysis requires a large number of analysis steps, but the basis function of the FRM uses only one-step static FE analysis. In the design optimization process of the auxiliary tooth, only one static FE analysis is performed to obtain the auxiliary tooth field information. Then, using the basis function, the overall air-gap magnetic flux distribution for the separation distance of the auxiliary tooth can be predicted with simple mathematical calculations without additional FE analysis. The introduced virtual air-gap section method is effectively used to analyze the non-linear characteristics of complex 3-D magnetic fields such as overhang structures.

It is a well-known fact that in multi-objective optimization, a compromise between performances is essential to select optimal design parameters. In the problem domain within the given requirements, this indicates that not only the global peaks of the objective function but also the local peaks can be an alternative as an optimal solution [18,19,20]. The Climb method is an efficient and practical multimodal optimization that requires fewer function evaluations compared to conventional approaches [21]. It constructs a level curve using an efficient interpolation method to estimate the problem domain and derives only one solution corresponding to each level group. However, this technique does not take into account the robustness evaluation of local peaks, which are used as indicators for selecting the optimal solution [22,23]. Based on the Climb method, the proposed optimization algorithm presents a technique to verify the robustness qualification in the process of searching the local peak without additional robustness evaluation.

The rest of this paper is organized as follows. In Section 2, using the proposed method based on the FRM, magnetic field analysis of the LPMM is presented. The armature reaction field and the open-circuit field are evaluated, and the air-gap magnetic flux distribution due to the auxiliary tooth is analyzed. The virtual air-gap section method is introduced for non-linear 3-D magnetic field analysis of overhang structures and PM skew. In Section 3, calculations of motor characteristics for the LPMM are provided, such as detent force, end force, thrust, and back-EMF. After that, the characteristics of the LPMM for combinations between auxiliary tooth thickness and separation distance from back-irons are presented along with comparative analysis. In addition, the effect of the auxiliary tooth and overhang structure on the PM skew is analyzed, and consequently, the optimal length fluctuation is found. In Section 4, proposed techniques for multimodal and robust optimization based on the Climb method are introduced. In Section 5, the validity of the proposed method is verified via FE and experimental results. Finally, Section 6 concludes this paper.

## 2. Magnetic Field Analysis

In the LPMM, an air-gap magnetic flux density is expressed as follows:

(1) $$B_{x}(t,x,y,z)=B_{x,O}(t,x,y,z)+B_{x,A}(t,x,y,z)$$

(2) $$B_{y}(t,x,y,z)=B_{y,O}(t,x,y,z)+B_{y,A}(t,x,y,z)$$

(3) $$B_{z}(t,x,y,z)=B_{z,O}(t,x,y,z)+B_{z,A}(t,x,y,z)$$

where *B_x_*, *B_y_*, and *B_z_* are the total air-gap magnetic flux density in the *x*-, *y*-, and *z*-directions, respectively. *B_O_* and *B_A_* are the air-gap magnetic flux densities in the open-circuit field and the armature reaction field, respectively. In the LPMM, the open-circuit field *B_O_* is the magnetic field generated only by the PM of the stator, and the armature reaction field *B_A_* is the magnetic field generated only by the winding current of the mover. The total air-gap magnetic flux density is expressed as the sum of the magnetic flux densities in these two magnetic fields.

In the FRM, each magnetic field, *B_O_* and *B_A_*, is calculated using the respective basis function. The basis function stores the reference magnetic flux distribution of each magnetic field obtained from the static FE analysis. This reference magnetic field is swept by the basis function to reconstruct the entire magnetic field. It considers the change of the magnetic flux distribution according to the positions of the mover and the stator. In the magnetic field reconstruction process, the air-gap magnetic flux distribution is predicted and calculated corresponding to a specific position of the mover or any excitation current. The magnetic field analysis using the FRM not only has high accuracy by using the static FE analysis, but also provides a dramatic reduction in computational burden by calculating the magnetic field at the entire mover position and arbitrary excitation current.

### 2.1. Armature Reaction Field

**Figure 2:**
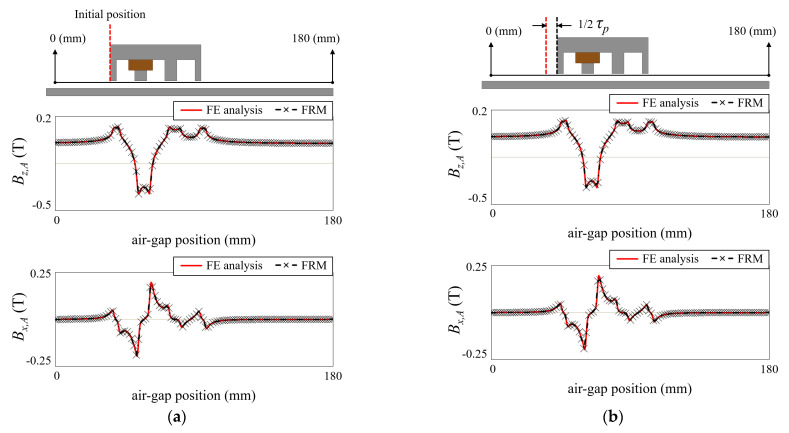

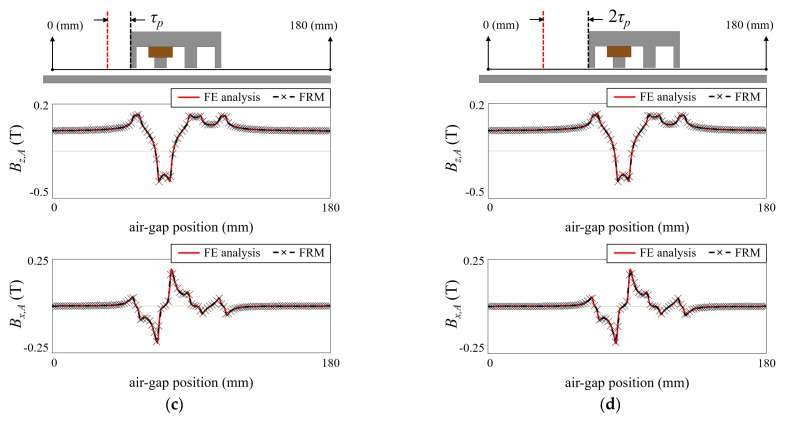
Armature reaction field of LPMM. (**a**) Mover is in initial position. (**b**) Moved by 1/2 *τ_p_*. (**c**) Moved by *τ_p_*. (**d**) Moved by 2 *τ_p_*.

In order to reconstruct the armature reaction field, a reference magnetic flux distribution should be obtained. This can be obtained by the static FE analysis with an initial DC current applied without the PM. Since the reference magnetic field contains structural information of the stator and the mover, it is necessary to consider how the magnetic flux distribution is affected by the mover position. Figure 2 shows the air-gap magnetic flux distribution according to the position of the mover in the armature reaction field. The PM is excluded, there is only the back-iron for the PM in the stator, and the initial DC current is applied to the second winding. In Figure 2a, the mover is positioned at the initial position, and in Figure 2b–d), the mover is positioned 1/2 *τ_p_*, *τ_p_*, and 2 *τ_p_* away from the initial position. *τ_p_* is the pole pitch. It is noted that the armature reaction field is maintained regardless of the position of the mover, as shown in Figure 2. This is because the reluctance across the effective air-gap does not change when the position of the mover changes. Therefore, the armature reaction field is reconstructed as follows:

(4) $$B_{A}(t,x,y,z)=\sum_{k=1}^{N_{S}}f_{A,k}\left(t,x,y,z,B_{A,ref.}^{}(x,y,z)\right)\cdot i_{k}\left(t\right)$$

(5) $$B_{A,ref.}(x,y,z)=\frac{B_{A}(x,y,z,t_{0},I_{0})}{I_{0}}\;$$

where *f_A,k_* is the basis function of the armature reaction field, *N_S_* is the number of slots, *i_k_* is the *k*th slot winding current, *B_A,ref._* is the reference magnetic flux distribution of the armature field, *t*_0_ is the initial time, and *I*_0_ is the initial current.

The total armature reaction field is expressed as the sum of the magnetic fields generated from the excitation currents of the individual slot windings. In the armature reaction field, since there is no waveform change according to the position of the mover, the reference magnetic flux distribution *B_A,ref._* can be obtained using the static FE analysis. The basis function sweeps the reference magnetic flux distribution to reconstruct the magnetic flux distribution at a specific mover position as follows:

(6) $$f_{A,k}\left(t,x,y,z\right)=f_{A,k}\left(t_{0},x+vt,y,z\right)$$

where *v* is the velocity of the LPMM.

### 2.2. Open-Circuit Field

**Figure 3:**
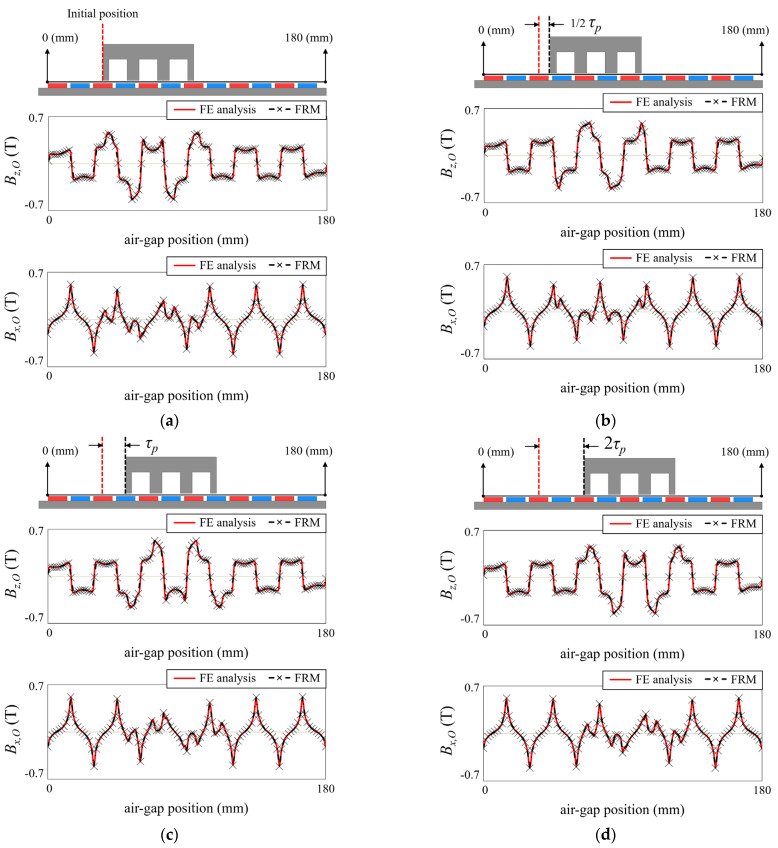
Open-circuit field of LPMM. (**a**) Mover is in initial position. (**b**) Moved by 1/2 *τ_p_*. (**c**) Moved by *τ_p_*. (**d**) Moved by 2 *τ_p_*.

On the other hand, in the open-circuit field generated by the PM, the waveform change should be considered according to the position of the mover. Figure 3 shows the open-circuit field at different mover positions. The movers are located in initial positions, 1/2 *τ_p_*, *τ_p_*, and 2 *τ_p_*, respectively. Unlike the armature reaction field, the open-circuit field repeats with a period of 2 *τ_p_*. This is because the effective air-gap reluctance changes with the position of the tooth of the mover. Therefore, field reconstruction using the basis function is performed as follows:

(7) $$B_{O}(t,x,y,z)=\sum_{h=1}^{N_{P}}f_{O,h}\left(t,x,y,z,B_{O,ref.}^{}(t,x,y,z)\right)$$

(8) $$f_{O,h}\left(t,x,y,z\right)=f_{O,h}\left(t+2\gamma \tau_{p}/v,x+2\gamma \tau_{p},y,z\right)$$

(9) $$\gamma =0,1,2,\ldots ,\left(L_{stroke}/(2\tau_{p})-1\right)\;$$

where *f_O,k_* is the basis function of the open-circuit field, *N_P_* is the number of PMs, *B_O,ref._* is the reference magnetic flux distribution of the open-circuit field, *γ* is the sweeping coefficient, and *L_stroke_* is the length of the stroke. The total open-circuit field is expressed as the sum of the magnetic fields generated by the individual PMs.

It is well-known that adding an auxiliary tooth at both ends of the back-iron is one of the effective ways to reduce detent force. However, there is a disadvantage that the characteristics change due to the distortion of the magnetic flux distribution in the air-gap. To solve this problem, a technique has been developed that inserts a non-magnetic material between the auxiliary tooth and the back-iron. This is to avoid creating loops in the magnetic flux path through the auxiliary tooth and back-iron. Therefore, the magnetic flux density inside the back-iron is maintained regardless of the presence or absence of an auxiliary tooth.

**Figure 4:**
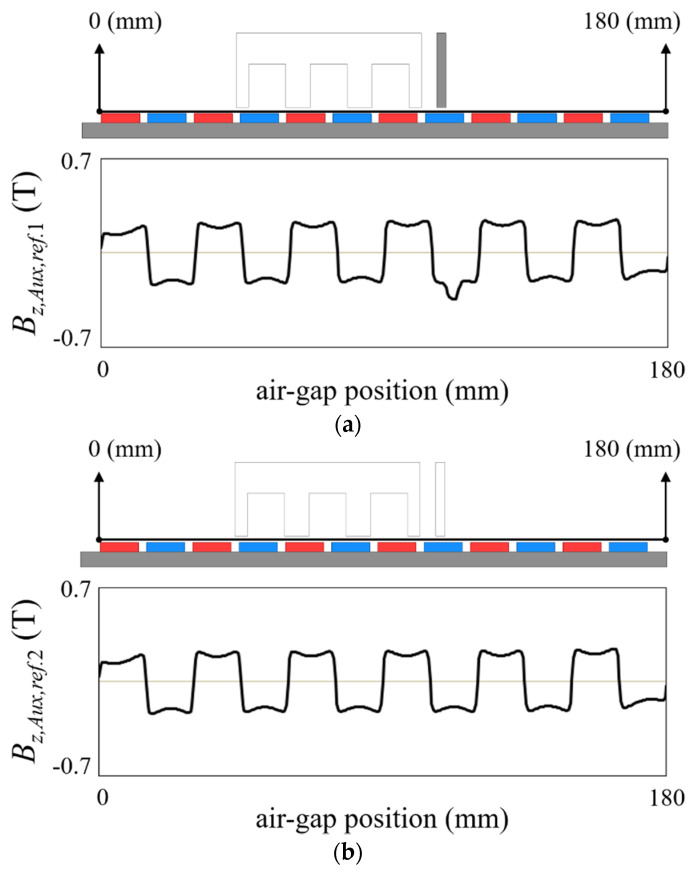

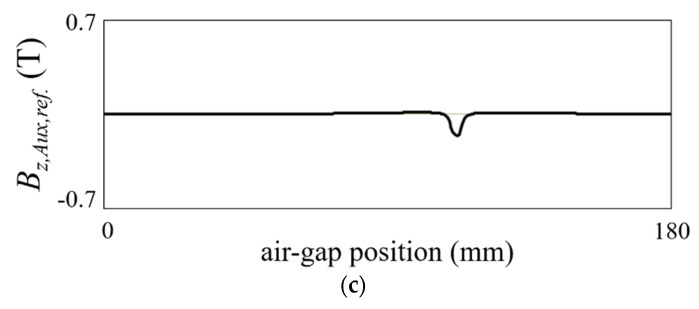
Reference magnetic flux distribution of auxiliary tooth. (**a**) *B_Aux,ref._*_1_. (**b**) *B_Aux,ref._*_2_. (**c**) *B_Aux,ref._*.

In the magnetic field analysis of the LPMM with an auxiliary tooth, since the auxiliary tooth and back-iron are isolated, the armature reaction field generated by the winding excitation current is constant with or without an auxiliary tooth. However, the effect of the auxiliary tooth on the open-circuit field should be analyzed and considered. The open-circuit field with the auxiliary tooth is expressed as

(10) $$B_{O}(t,x,y,z)=\sum_{h=1}^{N_{P}}f_{O,h}\left(t,x,y,z,B_{O,ref.}^{}(t,x,y,z)\right)+f_{Aux}\left(t,x,y,z,B_{Aux,ref.}^{}(t,x^{\prime },y^{\prime },z^{\prime })\right)$$

(11) $$B_{Aux,ref.}^{}(t,x^{\prime },y^{\prime },z^{\prime })=B_{Aux,ref.1}^{}(t,x^{\prime },y^{\prime },z^{\prime })-B_{Aux,ref.2}^{}(t_{0},x,y,z)\;$$

where *f_Aux_* is the basis function of the auxiliary tooth, *B_Aux,ref._* is the reference magnetic flux distribution of the auxiliary tooth, and *B_Aux,ref._*_1_ and *B_Aux,ref._*_2_ are the magnetic fields to make *B_Aux,ref._*, as shown in Figure 4. *B_Aux,ref._*_1_ is obtained through FE analysis with only the auxiliary tooth excluding the back-iron, and *B_Aux,ref._*_2_ is the air-gap magnetic flux distribution in the absence of both the back-iron and the auxiliary tooth. The air-gap magnetic flux distribution of the auxiliary tooth has a periodicity as much as 2 *τ_p_*, so it is swept as follows:

(12) $$f_{Aux}\left(t,x,y,z\right)=f_{Aux}\left(t+2\gamma \tau_{p}/v,x+2\gamma \tau_{p},y,z\right)$$

(13) $$x^{\prime }=x+AL$$

where *AL* is the separation distance of the auxiliary tooth from the back-iron. The separation distance and thickness of the auxiliary tooth from the back-iron are important design variables to reduce detent force, and the FRM relieves the dramatic computational burden in the process of finding these optimal design variables. The force calculation of the LPMM with an auxiliary tooth is introduced in the next chapter.

### 2.3. Virtual Air-Gap Section

In this paper, PM skew and an overhang structure are adopted to improve motor performance with reduction of detent force. PM skew is widely used to reduce the sum of the total detent force by phase shifting the magnetic flux distribution in the stack direction. In the case of the overhang structure, it is used to compensate for the relatively reduced magnetic flux density at the back-iron tip in the stack direction. For magnetic field analysis of an LPMM with PM skew and overhang structures, however, 3-D structural effects should be considered. These 3-D structures make the magnetic flux distribution in the air-gap non-uniform in the stack direction. 

**Figure 5:**
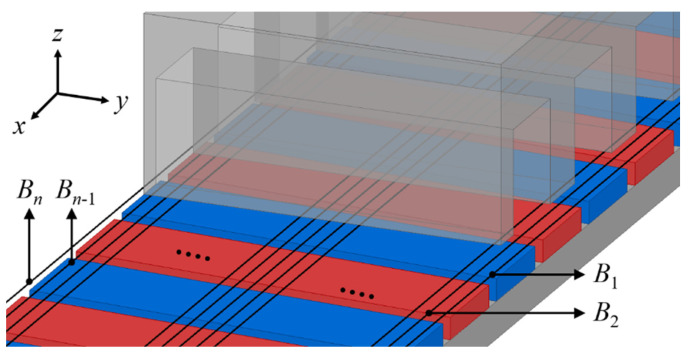
Virtual air-gap section.

Figure 5 shows the air-gap of the LPMM with PM skew and overhang structures. Each air-gap section has different magnetic flux distributions due to the phase shifting and leakage flux. These 3-D effects require a large amount of information to obtain the reference magnetic flux distribution for the basis function. The introduced virtual air-gap section is used to obtain the reference magnetic field in the air-gap composed of a non-uniform magnetic flux distribution as follows:

(14) $$B_{ref.}\left(x,y,z\right)=\frac{1}{n}\sum_{i=1}^{n}B_{i}\left(x,y_{i},z\right).$$

## 3. Comparative Study

**Figure 6:**
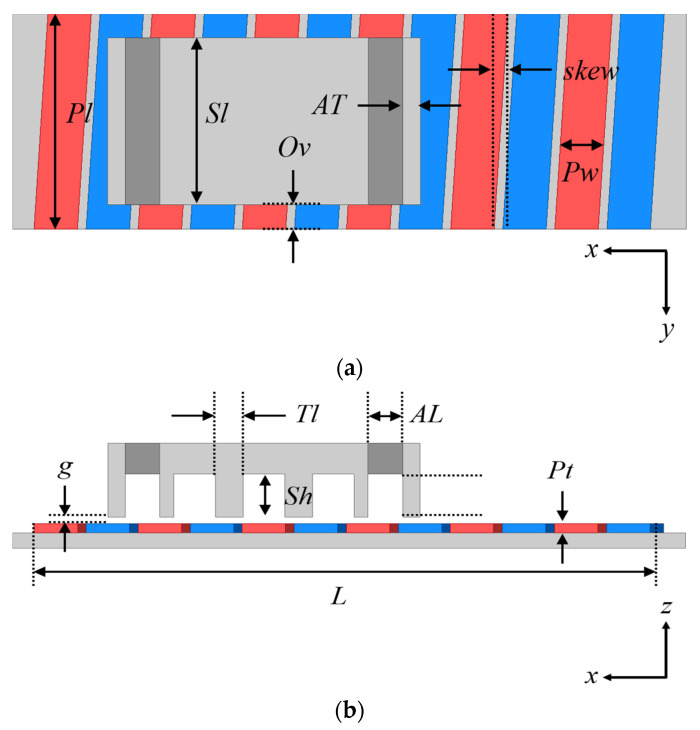
Design parameters. (**a**) Top view. (**b**) Side view.

In this section, a comparative analysis of effective detent reduction methods of LPMMs is provided. First, the detent force according to the combination of separation distance and thickness of auxiliary tooth is analyzed, and the minimum separation distance under load conditions that do not affect thrust are introduced. In LPMMs with an auxiliary tooth and overhang structure, analysis of the optimal length of PM skew is provided. The design parameters of the LPMM are shown in Figure 6 and Table 1. The basic model specifications for comparative studies are shown in Table 2.

### 3.1. Characteristic Calculation

In electrical machines, the detent force is due to the interaction between the back-iron of the mover and the PM of the stator. Unlike rotating machines, LPMMs have the end effect, which is a magnetic flux distortion that occurs at both ends of the mover back-iron. The force generated by these end effects is called the end force and is included in the detent force of the LPMM. Therefore, the detent force of the LPMM can be expressed as the sum of the end force and the slot effect force generated by the interaction between the slot and the PM.

In this paper, the force of the LPMM is calculated using the Maxwell stress tensor as follows:

(15) $$F=\int_{S}\overset{↔}{T}\cdot \hat{n}dS$$

where $\overset{↔}{T}$ is the Mexwell stress tensor, *S* is the surface of the air-gap, and $\hat{n}$ is the normal vector on the surface. 

**Figure 7:**
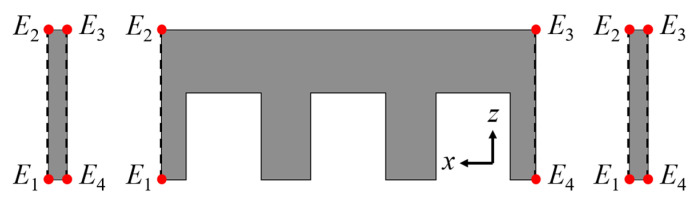
Side view of back-iron and auxiliary tooth.

Since the detent force is characteristic under no-load conditions, only the open-circuit field is required for calculation. First, the slot effect force is calculated as follows:

(16) $$F_{x}=\frac{1}{\mu_{0}}\int_{L}\int_{Pl}B_{x,O}B_{z,O}dxdy$$

where *L* is the air-gap length in moving direction, *Pl* is the PM length, and *μ*_0_ is the vacuum permeability. On the other hand, the end force is calculated as

(17) $$F_{x,end,left}=\frac{1}{\mu_{0}}\int_{L}\int_{E_{1}E_{2}}\left(B_{x,O}^{2}-B_{y,O}^{2}-B_{z,O}^{2}\right)dzdy$$

(18) $$F_{x,end,right}=\frac{1}{\mu_{0}}\int_{L}\int_{E_{3}E_{4}}\left(B_{x,O}^{2}-B_{y,O}^{2}-B_{z,O}^{2}\right)dzdy$$

(19) $$F_{x,end}=F_{x,end,left}+F_{x,end,right}$$

where *F_x,end,left_* and *F_x,end,right_* are the end forces generated at the left and right ends of the back-iron, respectively, and *E*_1_ to *E*_4_ are points for calculating the end force, shown in Figure 7. The total end force is expressed as the sum of these two forces. For the calculation of thrust, the total magnetic flux density is used, which is the sum of the open-circuit field and the armature reaction field as

(20) $$F_{x}=\frac{1}{\mu_{0}}\int_{L}\int_{}B_{x}B_{z}dxdy.$$

**Figure 8:**
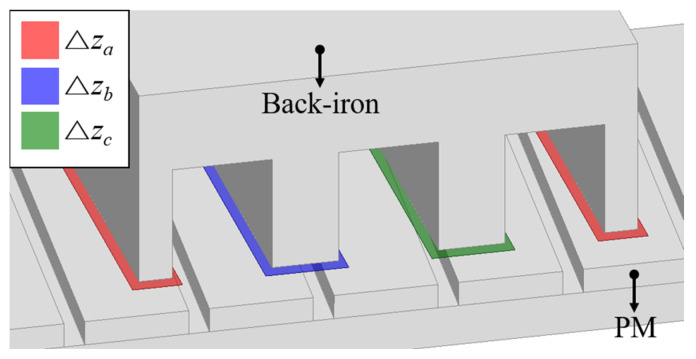
Virtual tooth plane.

The auxiliary tooth, which is isolated from the back-iron by non-magnetic material, does not affect the flux linkage. However, since the PM skew and overhang structure change the air-gap magnetic flux distribution, the effect on flux linkage and back-EMF should be considered. Considering that the magnetic flux loop in the LPMM is confined to the inside of the back-iron, most of the magnetic flux inside the back-iron passes through the tooth. Therefore, the flux linkage can be calculated as the magnetic flux density in the virtual tooth plane Δ*z_a_* in Figure 8. Since the magnetic flux from the virtual tooth plane to the back-iron is the *z*-direction component, the flux linkage is calculated as follows:

(21) $$\lambda_{a}\left(t\right)=N_{a}\iint_{\Delta z_{a}}\left(B_{z,O}(t)+B_{z,A}(t)\right)dxdy$$

where *λ_a_* and *N_a_* are the flux linkage and the number of turns in the *a*-phase winding, respectively. The flux linkage of the *a*-phase winding under no-load condition can be calculated using only the open-circuit field, and the *a*-phase back-EMF based on the voltage equation can be calculated as

(22) $$\lambda_{a,noload}\left(t\right)=N_{a}\iint_{\Delta z_{a}}B_{z,O}(t)dxdy$$

(23) $$e_{a}=\frac{d\lambda_{a,noload}}{dt}$$

where *e_a_* is the *a*-phase back-EMF.

**Figure 9:**
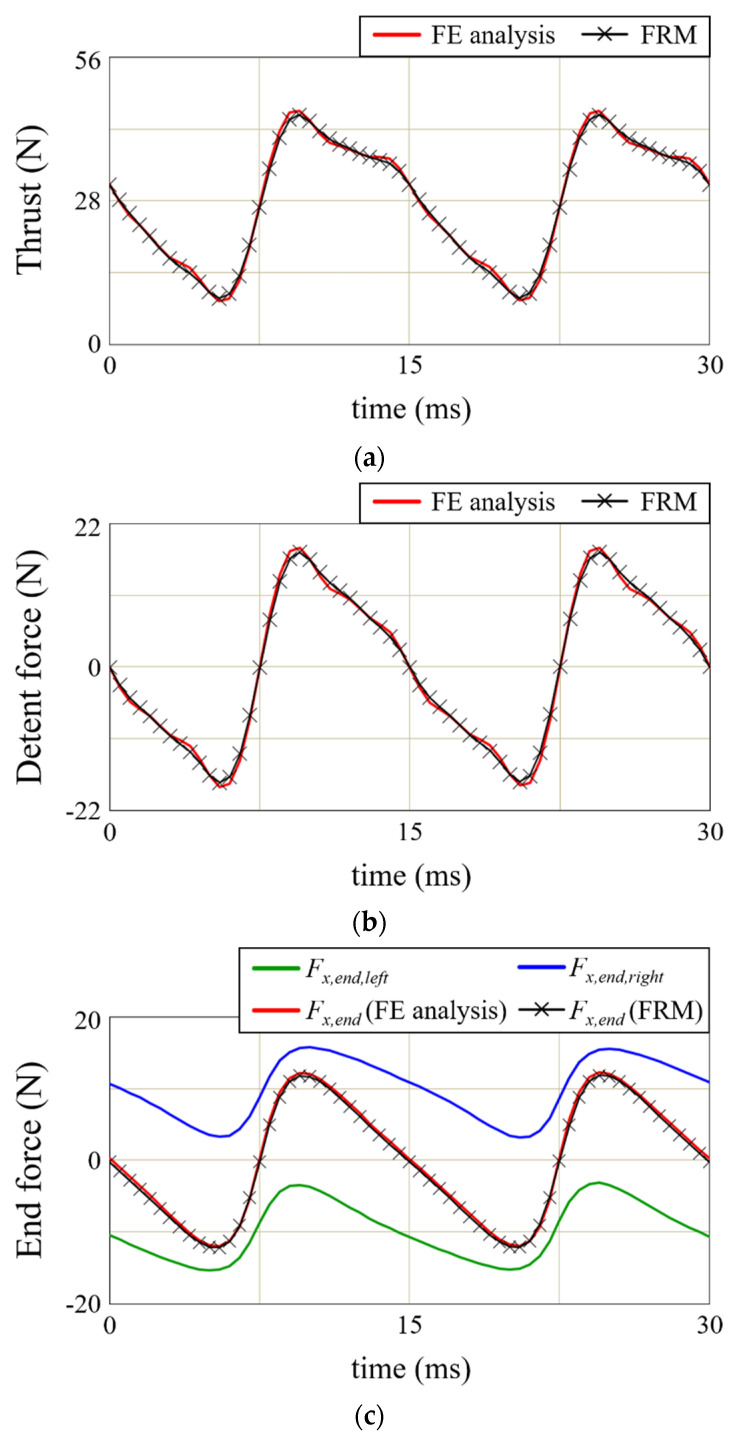
Force calculation of basic model. (**a**) Thrust. (**b**) Detent force. (**c**) End force.

Figure 9 shows the comparison of the force calculation of the basic model using the FRM with the FE analysis result. The thrust calculation in Figure 9a was calculated in Equation (20) using the total air-gap magnetic flux density, and Equation (16) was used for the calculation of the detent force in Figure 9b. The force results using the FRM have an acceptable difference of less than 5% from the FE analysis results. Figure 9c shows the end force of the LPMM in Table 2. The end force occurs at both ends of the mover’s back-iron and can be calculated using Equations (17)–(19). The total end force is expressed as the sum of *F_x,end,left_* and *F_x,end,right_*. It is added to the detent force to cause a ripple in thrust. As shown in Figure 9c, the end force always has a period of pole pitch.

### 3.2. Auxiliary Tooth Effects

The auxiliary tooth addition method is to reduce the end force to lower the overall detent force. Since the detent force generated by the auxiliary tooth also has a period of pole pitch, the detent force can be reduced in the phase opposite to the end force.

**Figure 10:**
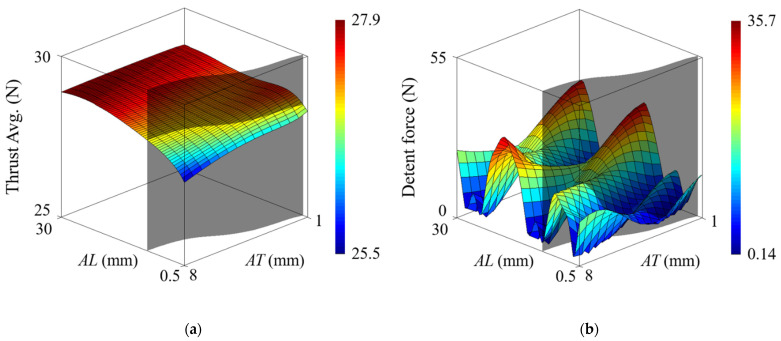
Auxiliary tooth effect. (**a**) Average thrust. (**b**) Maximum detent force.

The separation distance and thickness of the auxiliary tooth are important factors for reducing detent force. Figure 10 shows the average thrust and maximum detent force according to the combination of separation distance *AL* and thickness *AT*. The auxiliary tooth adopted in this paper is isolated from the back-iron to prevent distortion of load characteristics by making the magnetic flux inside the back-iron not pass through the auxiliary tooth. However, it is noted that the average thrust decreases when the separation distance is shorter than a certain length, as shown in Figure 10a. This means that minimum separation distance should be considered to prevent magnetic flux passing to the auxiliary tooth. The minimum separation distance *AL* increases as the thickness *AT* of the auxiliary tooth increases.

In Figure 10b, considering that the maximum value of the detent force of the basic model is 17.6 N, the minimum value of the detent force with the auxiliary tooth is 0.14 N, which results in a 99.2% reduction. On the other hand, the maximum value is 35.7 N, which causes an increase of 102.8%. This is because the phase of the force generated by the auxiliary tooth is the same as that of the end force. Furthermore, it is shown that the detent force for the combination of *AL* and *AT* changes irregularly, and there are various options for the optimal solution. Therefore, in the process of optimizing the auxiliary tooth, an appropriate thickness and separation distance should be selected, as well as the minimum separation distance related to distortion of load characteristics.

### 3.3. PM Skew with Auxiliary Tooth

The optimal PM skew used in the rotating machine is calculated based on the relationship between the number of slots and the number of poles. This is because the no-load force has a period corresponding to the least common multiple of the number of slots and the number of poles. In the LPMM, since the detent force is composed of the sum of the end force and the slot effect force, the optimal skew length is determined depending on the detent force waveform. The harmonic component of the total detent force can be a measure of the period of the waveform and contributes to determining the appropriate optimal PM skew length.

As the end force decreases due to the auxiliary tooth, the ratio of the slot effect force to the total detent force increases. However, when the *AL* is in phase with the end force, the total detent force increases further.

**Figure 11:**
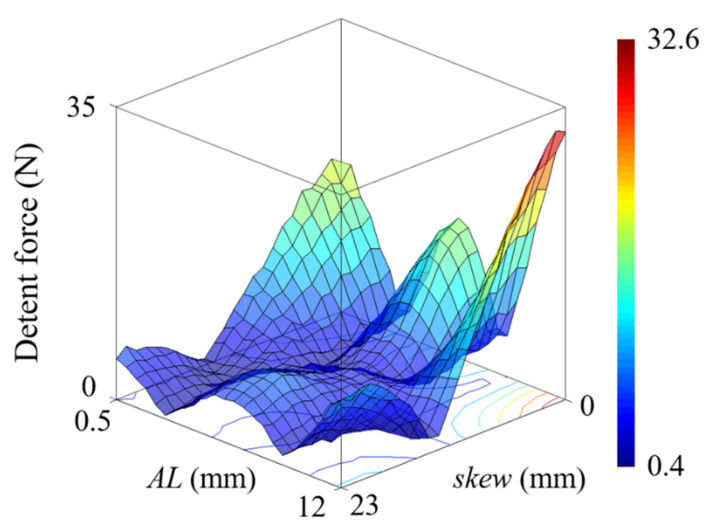
Maximum detent force for separation distance and PM skew length.

**Figure 12:**
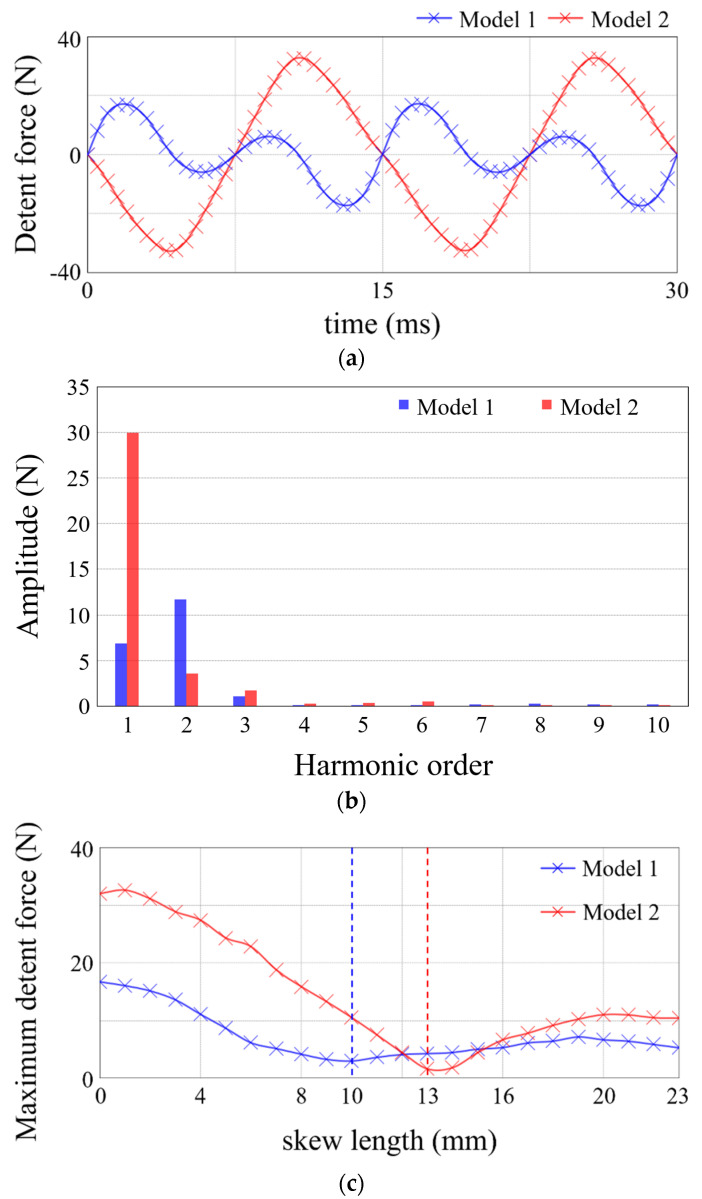
Force calculation of basic model. (**a**) Thrust. (**b**) Detent force. (**c**) End force.

Given that the period of the slot effect force and the end force is different, the optimal skew length is determined by the contribution of these two forces to the total detent force. Figure 11 shows the maximum value of the detent force according to the combination of the separation distance *AL* and *skew*, where *AT* is 1.2 mm. In the LPMMs with different *AL*s, the optimal skew length is not the same. Table 3 compares two models with an *AL* of 6.5 mm and 12 mm, respectively. The detent forces of model 1 and model 2 are compared as in Figure 12a. For both detent forces, the result of the fast Fourier transform is shown in Figure 12b. In the case of model 1, it is confirmed that the amplitude of the 133.3 Hz component is the largest among the harmonic components of the waveform. On the other hand, for model 2, the 66.7 Hz component stands out among the total harmonics. Therefore, the length of PM skew required to minimize detent force is smaller in model 1 than in model 2. Figure 12c shows the maximum detent force for the PM skew length in both models. It is noted that the optimal PM skew length for model 1 is 10 mm, while for model 2 it is 13 mm. In model 1 and model 2, the maximum detent forces under the condition of no PM skew are 16.7 N and 32.0 N, respectively, and 3.0 N and 1.6 N when the optimal PM skew of each model is applied. By using the PM skew, the detent force of both models was reduced by 82.0% and 94.8%, respectively.

### 3.4. PM Skew with Overhang Structure

The LPMM has flux leakage at the stack direction end, which causes a decrease in the overall magnetic flux density. The overhang structure is a technique to increase the PM length to compensate for this leakage flux. However, when using an overhang structure and PM skew at the same time, there is one thing to consider: the change in optimal PM skew length.

**Figure 13:**
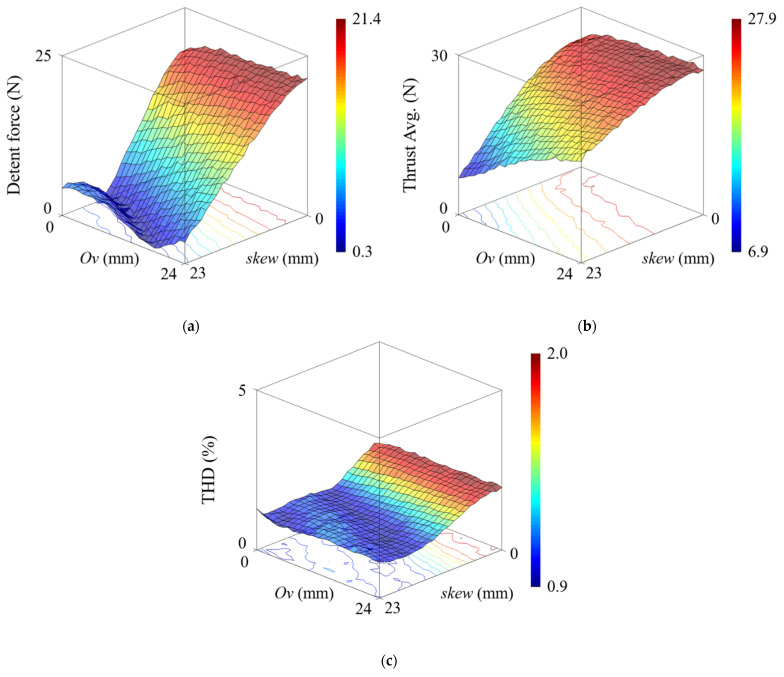
Comparative analysis result of overhang length and PM skew length. (**a**) Maximum detent force. (**b**) Average thrust. (**c**) THD.

Figure 13 shows the characteristics of the LPMM according to the combination of overhang length and PM skew. The LPMM is a basic model, with the specifications in Table 1, and the auxiliary tooth is excluded. Figure 13a shows the maximum value of detent force according to the combinations. The highest and lowest values of detent force are 21.4 N and 0.3 N, respectively, providing a 98.6% reduction. It is noted that the optimal skew length at which the detent force is minimized is different depending on the overhang length *Ov*.

**Figure 14:**
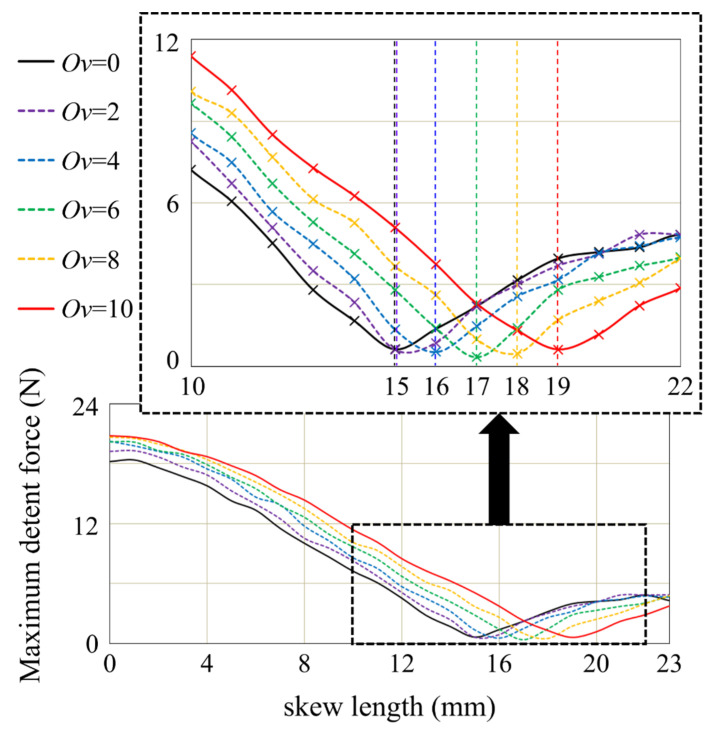
Maximum detent force for PM skew length of LPMM with different overhang length.

Figure 14 shows the maximum detent force for skew lengths of LPMMs with different overhang lengths. For non-overhang models, the skew length of the model with the minimum detent force is 15 mm. However, for the LPMM with an overhang length of 10 mm, the optimal skew length is found to be 19 mm. As shown in Figure 14, as the overhang length increases, the optimal skew length also increases. This is because the effective air-gap corresponding to the back-iron length has a skew effect that is smaller than the actual skew length, so a longer skew length is required.

Figure 13b,c show the average thrust and THD of the EMF according to the combination of overhang length and skew length, respectively. The average thrust was calculated using Equation (20), and the EMF calculated in Equation (23) was used for the THD. It can be seen that the overhang structure contributes to the thrust improvement but is saturated. This is because either the air-gap magnetic flux density in the overhang region is saturated or the distance between the PM and the back-iron is too far away so that the magnetic flux deviates from the path. Consequently, for the overhang structure, it is necessary to select an appropriate overhang length and determine the optimal PM skew length accordingly.

## 4. Optimization

In multi-objective optimization involving non-linear effects, design parameters are determined as a compromise between motor performances. For some specific objective functions, the trade-off often results in a local optimal rather than a global optimal being selected as the optimal solution. The multimodal optimization provides designers with alternative solutions in the problem domain within their requirements. The Climb method is the multimodal optimization using contour lines [21]. Unlike conventional optimization techniques, where the validity check of local peaks is performed when a convergence condition is reached, in this method, it is performed simultaneously during the process of finding local peaks. Therefore, it has the advantage of quickly and effectively searching the global optimal and the local optimal, especially in an objective function with a large problem domain or many optimal solutions. In the Climb method, however, the evaluation of robustness is not taken into account, an important factor for evaluating the qualifications of local peaks. The proposed algorithm provides a novel strategy for effective robustness evaluation with multimodal optimization based on the Climb method.

**Figure 15:**
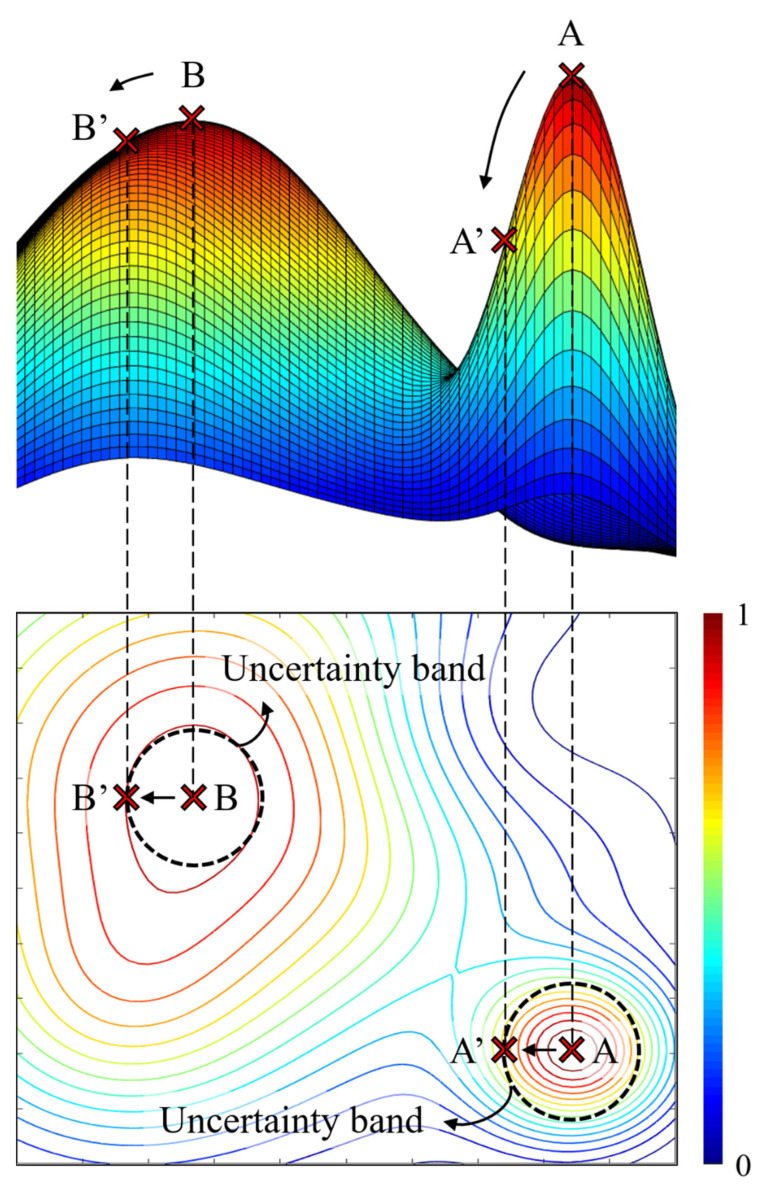
Uncertainty bands in problem domain.

In the problem domain in Figure 15, it is confirmed that the global peak is point A. It is clear that point A will be the optimal solution under the condition that the accuracy of the design parameters is guaranteed. However, in the worst case, when the parameter moves from point A to point A′ above the uncertainty band, point A loses its qualification as a global peak. In Figure 15, the contour level corresponding to the desired value can be used as a decision maker to verify the robustness of the local peak inside the contour. As precise design is required, the robustness evaluation of local peaks is an important factor for selecting the optimal solution and should be considered in the multimodal optimization.

**Figure 16:**
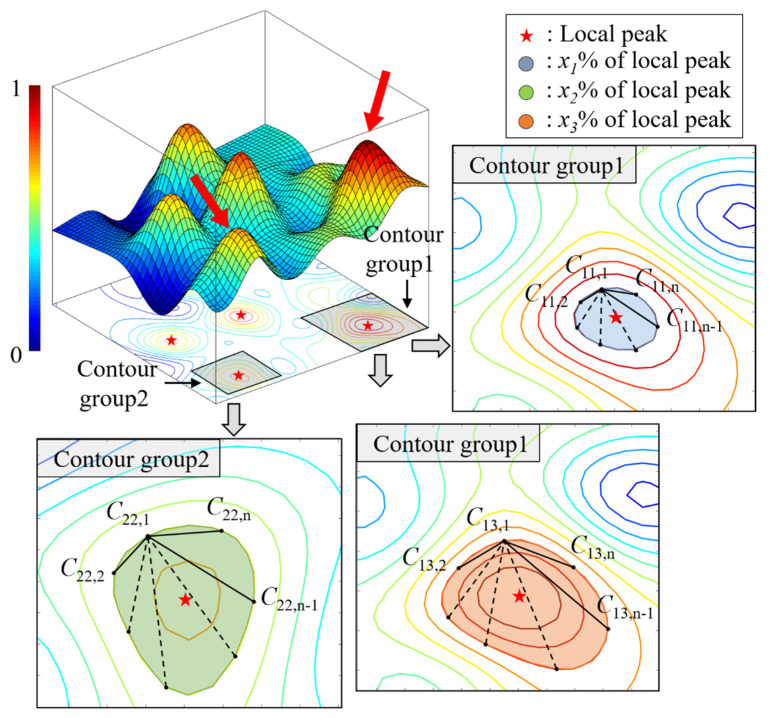
Robustness evaluation in surrogate model.

Figure 16 shows the surrogate model and local peaks of an objective function constructed using the Climb method. The detailed process involved in the construction of the surrogate model and the searching of local peaks is described in [21]. The contour lines corresponding to the level ratio of the local peak consist of point *C_kp,n_*, where *C_kp,n_* is the *n-*th point constituting the *p-*th contour in the *k-*th contour group. The area at the contour level represents the robustness range of the internal local peak from the uncertainty parameter at the desired ratio value *x*.

**Figure 17:**
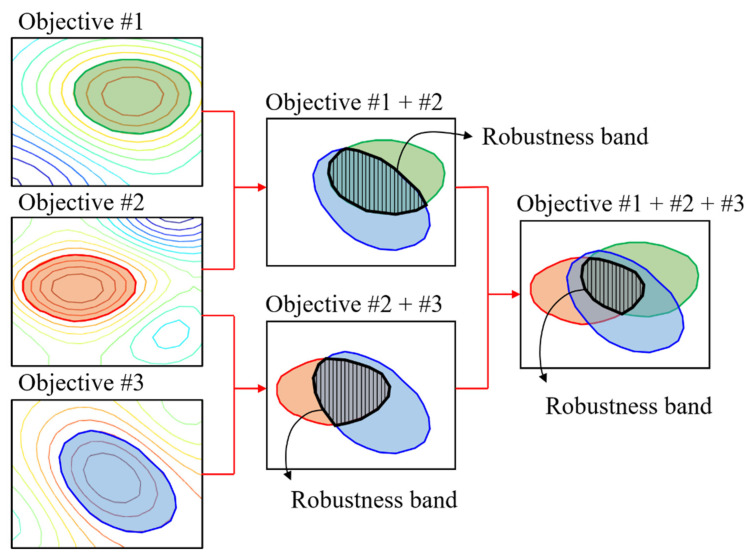
Robustness evaluation in multi-objective optimization.

In multi-objective optimization, since the contour groups have the robustness information of each local peak for the desired performance level value, the optimal solution can be derived by comparing the robustness bands in Figure 17. Since the additional robustness evaluation process is omitted, the computational burden is significantly reduced, especially in iterations in multi-objective optimization.

## 5. Result

**Figure 18:**
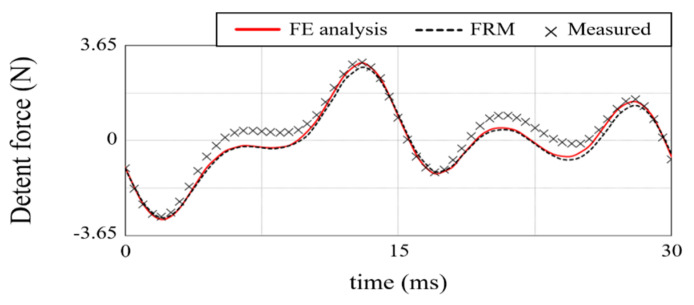
Detent force.

**Figure 19:**
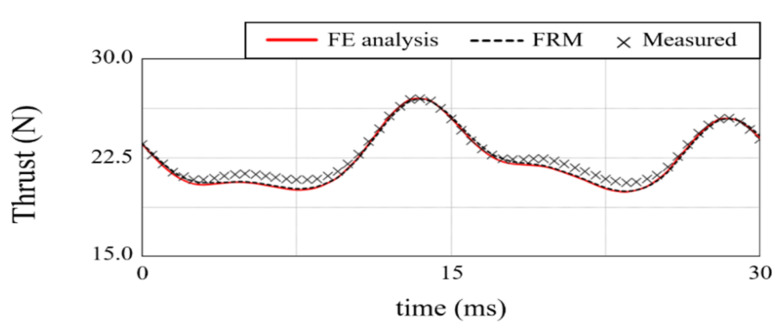
Thrust.

In this paper, JMAG, a well-known magnetic field analysis tool, was used for the FE analysis. The PM overhang structure and skew have complex 3-D structures that are not available for 2-D FE analysis. This results in a high time cost burden as it significantly increases the number of elements that need to be analyzed. To evaluate one cycle of force in Figure 18 and Figure 19, iterations of transient analysis are performed with a sufficient number of time steps. In the FRM, however, the armature reaction field requires only one time-step static FE analysis, and the properties corresponding to the remaining time-steps are reconstructed using a mathematical sweep of the basis function. Therefore, the FRM provides significant computational burden reduction compared to the conventional transient 3-D FE analysis. In particular, the optimization process of the auxiliary teeth using the FRM has the advantage of being performed as a theoretical approach through the basis function without additional FE analysis.

Table 4 describes the test model conditions. The design parameters are based on the basic model in Table 2. Each design parameter was selected by performing an optimization process. We aimed to have LPMMs with features such as low detent force and low THD along with high thrust. Using the characteristic calculation in Chapter 3, we obtain the characteristic results for AT and AL, which are the variables of the auxiliary tooth, and the characteristics according to the combination between the PM skew length and the auxiliary tooth variables. At this time, among the combinations of auxiliary tooth variables, the case where the auxiliary tooth reduced the average force by more than 1.9% was excluded, as shown in Figure 10. Next, we obtain the values of the properties according to the combination of overhang length and PM length. These are mapped as contour lines via the Climb method within the problem domain. In the multi-objective optimization process, local peaks corresponding to each characteristic are searched and an robustness evaluation is performed simultaneously. The design parameters were determined with an appropriate compromise between combinations within the robustness band range between characteristics. On the mover, SS400 was used for the back-iron and auxiliary tooth. The non-magnetic material SUS304 is inserted between the back-iron and the auxiliary tooth to prevent distortion of the thrust. The thickness and separation distance of the auxiliary tooth were 1.2 mm and 8.0 mm, respectively, and the magnetic flux passing from the back-iron was considered. In the stator, the back-iron is SS400, with 4.0 mm overhang and 9.5 mm PM skew length.

**Figure 20:**
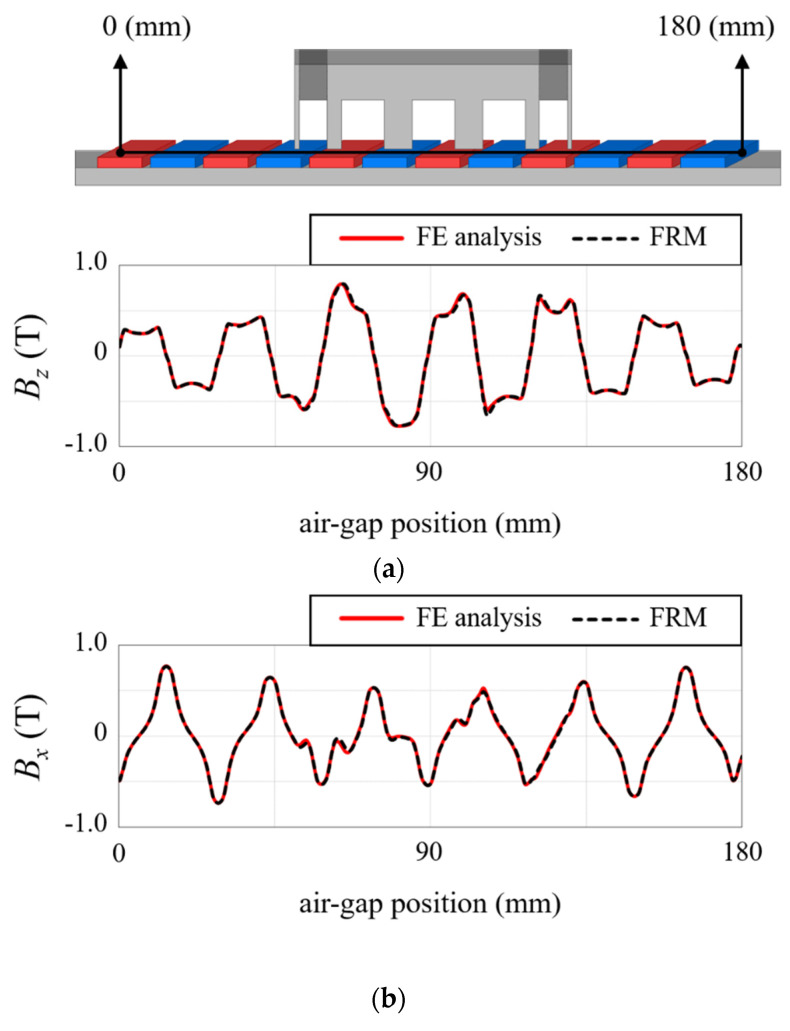
Air-gap magnetic flux distribution. (**a**) *z*-direction. (**b**) *x*-direction.

**Figure 21:**
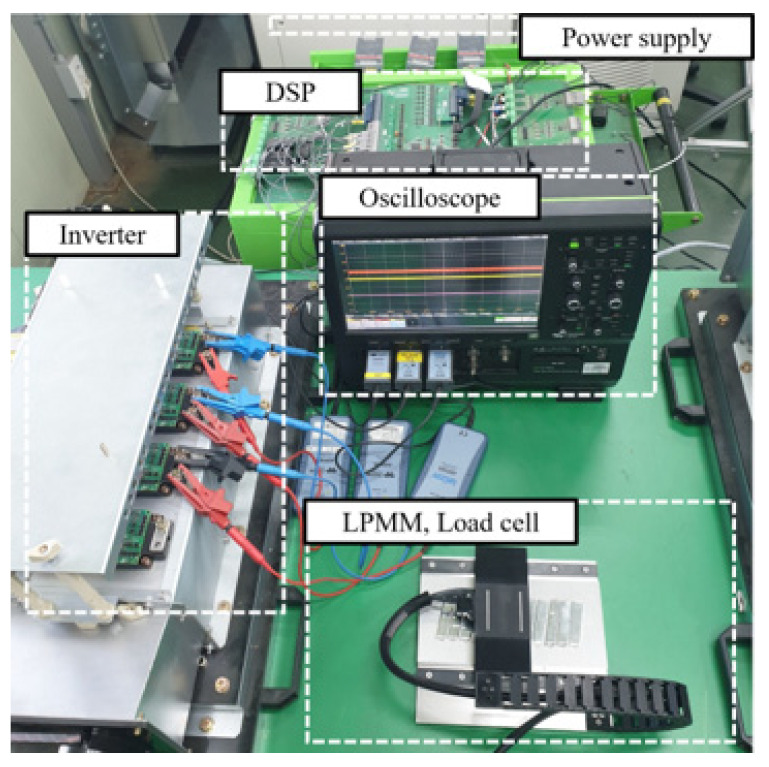
Test set.

Figure 20 compares the air-gap magnetic flux distribution reconstructed by the FRM with the FE analysis result. Figure 20a,b are *B_z_* and *B_x_*, respectively, with acceptable errors of less than 3%. Figure 21 shows the test setup. In order to accurately measure the thrust waveform, a load cell and a linear encoder were used instead of an acceleration sensor. TELEDYNE LECROY, CAS, and HEIDENHAIN products were used for the oscilloscope, load cell, and linear encoder, respectively. The load cell detects the force according to the position of the mover, and the digital indicator provides the equivalent thrust. For thrust calculation, the mover is set at the reference position where the EMF is zero. The excitation current is determined according to the movement distance from the reference position. The linear encoder was used to evaluate the exact position of the mover. Figure 18 shows the comparison result of detent force. The result calculated by the FRM was 3% different from the FE 3-D analysis result, and the difference from the measurement result was less than 5%. In the case of thrust, it is compared as shown in Figure 19. The results of the FRM differed from those of the FE analysis and measurement by 4% and 5%, respectively.

The FRM is a highly effective technique in terms of not only providing as high accuracy as FE 3-D analysis results, but also reducing the computational burden. In the thrust calculation process in Figure 19, the FE 3-D analysis took 26 m 39 s. Simulation conditions are CPU Intel core i7-11700K, GPU RTX 3060 Ti, RAM 32GB, Elements 186392. Under the same conditions, the FRM required only 1 m 31 s, which provided a 94.3% time cost reduction.

## 6. Conclusions

The main purpose of this paper is to effectively characterize models with complex 3-D structures, and the proposed magnetic field analysis method reduces the time burden by 94.3% compared to the conventional 3-D FE analysis. Using this method, it was investigated whether the optimal PM skew length varies with an auxiliary tooth, which was not considered in previous research. Additionally, it was investigated whether the PM overhang structure also affects the optimal PM skew length, and an optimization algorithm that can be used for the determination of the PM skew length is proposed.

This paper presents an LPMM with PM skew and an auxiliary tooth and overhang structure. The proposed magnetic field analysis method to analyze these complex 3-D effects dramatically reduces the computational burden but provides high accuracy. Using the basis function, the air-gap magnetic flux distribution can be predicted at a specific position of the mover and at an arbitrary excitation current. Especially, the change of magnetic field according to the separation distance and thickness of the auxiliary tooth was analyzed via the basis function without additional FE analysis. The non-uniform magnetic flux distribution in the stack direction due to PM skew and the overhang structure was calculated using the virtual air-gap section method.

The characteristics of the LPMM such as thrust, detent force, and flux linkage were calculated using the reconstructed magnetic field. The end effect, the cause of the end force, was investigated from both ends of the back-iron.

In the comparative analysis result, it should be noted that the minimum separation distance of the auxiliary tooth, which does not affect the load characteristics, varies according to the auxiliary thickness. Furthermore, depending on the combination of the separation distance and thickness, the detent force can be significantly reduced, but can be greater in certain cases. Therefore, in order to determine the optimal PM skew length, the harmonic components of the detent force should be considered. The effect of the overhang structure on the optimal PM skew length was investigated. As the overhang length increases, the optimal PM skew length should be increased because the effective skew length becomes shorter.

Finally, an effective multi-objective algorithm based on the Climb method is proposed. It not only finds local peaks quickly and accurately in multimodal optimization, but also applies a robustness evaluation that is not covered by the Climb method. The proposed algorithm has the advantage of significantly reducing the time cost because the robustness evaluation is performed simultaneously in the process of finding local peaks.

Among the techniques for motor improvement presented in this paper, the PM skew has the advantage of reducing the detent force, the addition of auxiliary teeth provides the reduction effect of the end force, and the PM overhang structure improves the force. Compared to rotary machines, the PM skew in the LPMM has good manufacturability, but still has the disadvantage of increasing the production cost for PM molding. In addition, since the auxiliary teeth increases the total volume, the thrust density per volume decreases, and the acceleration performance due to the increase in mass eventually decreases. The PM overhang structure has the advantage of increasing the maximum force within the limited capacity of the inverter and also has the disadvantage of increasing the overall manufacturing cost due to the increase in the amount of PM used. In particular, in the rotating machine, the stator and the rotor are abutted in a 1:1 ratio along the air-gap, whereas in the LPMM, the PM increase is higher for the overhang structure because the length of the PM is much longer than that of the mover. Of these technologies, the most expensive to manufacture are listed in the following order: PM overhang structure, auxiliary teeth, and PM skew.

In this paper, in the optimization of the auxiliary teeth, the traditional technique of adjusting thickness and separation distance is used. Recent papers related to auxiliary teeth suggest new shapes, such as shortening the height or forming rounded ends. These methods can be considered for future research as they are claimed to be more effective than traditional auxiliary teeth.

## Figures and Tables

**Table 1:** Design parameter of LPMM.

Parameter	Symbol
PM length (mm)	*Pl*
PM width (mm)	*Pw*
PM thickness (mm)	*Pt*
Slot length (mm)	*Sl*
Slot height (mm)	*Sh*
Tooth length (mm)	*Tl*
Overhang length (mm)	*Ov*
Air-gap (mm)	*g*
PM skew length (mm)	*skew*
Air-gap length (mm)	*L*
Auxiliary tooth thickness (mm)	*AT*
Auxiliary tooth separation distance (mm)	*AL*

**Table 2:** Specification of basic model.

Parameter	Value
PM length *Pl* (mm)	48
PM width *Pw* (mm)	12.5
PM thickness *Pt* (mm)	3
Slot length *Sl* (mm)	48
Slot height *Sh* (mm)	14
Tooth length *Tl* (mm)	8
Air-gap *g* (mm)	1
Air-gap length *L* (mm)	180
Current *I*_peak_ (A)	2
Motor speed *v* (m/s)	1

**Table 3:** Characteristics of LPMMs with different *AL*s.

Parameter	Model 1	Model 2
AT	1.2 mm	1.2 mm
AL	6.5 mm	12 mm
Optimal PM skew length	10 mm	13 mm
Maximum detent force (no skew)	16.7 N	32.0 N
Maximum detent force (skew)	3.0 N	1.6 N

**Table 4:** Test model condition.

Parameter	Value
Auxiliary tooth thickness, *AT* (mm)	1.2
Auxiliary tooth separation distance, *AL* (mm)	8.0
Overhang length, *Ov* (mm)	4.0
PM skew length, *skew* (mm)	9.5
Current, *I* (A)	2.0
Motor speed, *v* (m/s)	1.0
Frequency (Hz)	33.3

## Data Availability

Not applicable.
